# Maternal supply of cysteamine alleviates oxidative stress and enhances angiogenesis in porcine placenta

**DOI:** 10.1186/s40104-021-00609-8

**Published:** 2021-08-10

**Authors:** Shuangbo Huang, Zifang Wu, Zihao Huang, Xiangyu Hao, Longmiao Zhang, Chengjun Hu, Jianfu Wei, Jinping Deng, Chengquan Tan

**Affiliations:** 1 Guangdong Provincial Key Laboratory of Animal Nutrition Control, National Engineering Research Center for Breeding Swine Industry, Institute of Subtropical Animal Nutrition and Feed, College of Animal Science, South China Agricultural University, Guangzhou, 510642 Guangdong China; 2 Guangzhou DaBeiNong Agri-animal Huabandry Science and Technology Co., Ltd., Guangzhou, 510642 Guangdong China; 3 Guangdong Laboratory for Lingnan Modern Agriculture, South China Agricultural University, Guangzhou, 510642 Guangdong China

**Keywords:** Angiogenesis, Cysteamine, Oxidative stress, Placenta, Sow

## Abstract

**Background:**

Oxidative stress in placenta is associated with the occurrence of adverse pregnancy outcomes in sow, but there are few satisfactory treatment strategies for these conditions. This study investigated the potential of cysteamine (CS) as an antioxidant protectant for regulating the reproductive performance, redox status, and placental angiogenesis of sows.

**Methods:**

The placental oxidative stress status and vascular density of piglets with different birth weights: < 1.0 kg (low birth weight, LBW) and 1.4–1.6 kg (normal birth weight, NBW) were evaluated, followed by allotting 84 sows to four treatments (*n* = 21) and feeding them with a basal diet supplemented with 0, 100, 300, or 500 mg/kg of CS from d 85 of gestation to d 21 of lactation, respectively. Placenta, serum, and colostrum samples of sows or piglets were collected, and the characteristics of sows and piglets were recorded. Furthermore, the *in vivo* results were validated using porcine vascular endothelial cells (PVECs).

**Results:**

Compared with the NBW placentae, the LBW placentae showed increased oxidative damage and were vulnerable to angiogenesis impairment. Particularly, H_2_O_2_-induced oxidative stress prompted intracellular reactive oxygen species generation and inhibited the tube formation and migration of PVECs as well as the expression of vascular endothelial growth factor-A (VEGF-A) *in vitro*. However, dietary CS supplementation can alleviate oxidative stress and improve the reproductive performance of sows. Specifically, compared with the control group, dietary 100 mg/kg CS could (1) decrease the stillbirth and invalid rates, and increase both the piglet birth weight in the low yield sows and the placental efficiency; (2) increase glutathione and reduce malondialdehyde in both the serum and the colostrum of sows; (3) increase the levels of total antioxidant capacity and glutathione in LBW placentae; (4) increase the vascular density, the mRNA level of *VEGF-A*, and the immune-staining intensity of platelet endothelial cell adhesion molecule-1 in the LBW placentae. Furthermore, the *in vitro* experiment indicated that CS pre-treatment could significantly reverse the NADPH oxidase 2-ROS-mediated inactivation of signal transducer and activator of transcription-3 (Stat3) signaling pathway induced by H_2_O_2_ inhibition of the proliferation, tube formation, and migration of PVECs. Meanwhile, inhibition of Stat3 significantly decreased the cell viability, tube formation and the VEGF-A protein level in CS pretreated with H_2_O_2_-cultured PVECs.

**Conclusions:**

The results indicated that oxidative stress and impaired angiogenesis might contribute to the occurrence of LBW piglets during pregnancy, but CS supplementation at 100 mg/kg during late gestation and lactation of sows could alleviate oxidative stress and enhance angiogenesis in placenta, thereby increasing birth weight in low yield sows and reducing stillbirth rate. The *in vitro* data showed that the underlying mechanism for the positive effects of CS might be related to the activation of Stat3 in PVECs.

**Supplementary Information:**

The online version contains supplementary material available at 10.1186/s40104-021-00609-8.

## Introduction

Pregnancy is an oxidative stress challenge for the mother, especially during the late gestation period [1], when the rapid development of the fetus increases the metabolic burdens on pregnant sows or dams, leading to elevated systemic oxidative stress [2, 3]. Increasing evidence indicates that maternal oxidative stress is associated with several adverse outcomes, such as gestational diabetes mellitus, proteinuria preeclampsia, postpartum hemorrhage, fetal death, and low birth weight [4, 5]. Thus, reducing oxidative stress is a crucial issue for improving the reproductive efficiency of both humans and mammalian animals, but its underlying mechanisms still remain elusive.

The placenta regulates fetal nutritional and hormonal support, which, to a large degree, has a direct impact on the pregnancy outcome [6]. In particular, placental blood vessels play an important role in maternal-fetal material exchange, suggesting the importance of proper placental vasculature development for fetal growth. Moreover, the placenta is highly sensitive to oxidative stress [7], which could cause vascular dysfunction in the placenta [8, 9]. Our previous studies have uncovered that increased oxidative stress induced by maternal obesity may decrease the development of placental vasculature essential for fetal growth during pregnancy [10], suggesting the involvement of placental oxidative stress in the development of adverse pregnancy outcomes. Mechanically, an increased amount of reactive oxygen species (ROS) could induce autophagy, dysfunction, and apoptotic death of vascular endothelial cells [9]. The foregoing reports indicate that targeting the placenta could be an attractive strategy for modulating oxidative stress-related pregnancy diseases.

Dietary intake of antioxidant nutrients to improve the endogenous antioxidant defense capacity has been considered a plausible way to prevent oxidative stress [6, 11, 12]. Cysteamine (CS), a precursor of glutathione (GSH), is widely used to protect a series of tissues and organs such as gastrointestinal tract [13], brain [14], and kidney [15] from oxidative stress under adverse conditions. We selected CS based on its multiple beneficial modes of action, i.e., CS could improve ileal mucosal health by regulating the oxidation status and apoptosis in finishing pigs [13], and L-cysteine, one of the downstream metabolites of CS could efficiently reduce the inflammatory response in the maternal-fetal interface and improve the placental efficiency in rats [16]. In this regard, CS appears to be a promising candidate for alleviating maternal and placental oxidative stress. However, no data are available currently regarding the effects of dietary CS supplementation during gestation and lactation on the reproductive performance and antioxidant status of sows, especially the dose-effect relationship.

In this study, we addressed the hypothesis that CS could enhance placental angiogenesis by alleviating maternal and placental oxidative stress, and ultimately benefit the survival and growth of the fetus. Thus, the purpose of this study was to evaluate the effects of CS on sows’ reproductive performance, antioxidant status, and placental angiogenesis through an *in vitro*-*in vivo* method.

## Materials and methods

### Animals and experimental design

This study was conducted in Guangzhou DaBeiNong Agri-animal Huabandry Science and Technology Co., Ltd. A total of 84 Landrace × York sows (parities 2–5) were allocated to four dietary treatment groups with each sow as a replicate in a completely randomized design using body weight at d 85 of gestation as a block (*n* = 21 per treatment). The sows in the control group (CON) received a basal gestation or lactation diet without added CS from d 85 of gestation to d 21 of lactation, while the sows in the CS group were fed a basal diet supplemented with 100, 300, or 500 mg/kg of CS (CS100, CS300, and CS500 diet). All diets were formulated to meet the National Research Council (NRC, 2012) requirements of nutrient standards for gestational and lactational sows. The ingredients and compositions of the basal diet are shown in Supplementary Table S1.

Sows were housed in individual stalls and fed twice (07:30 and 17:00) a d with a constant amount of 3 kg during late gestation. During the entire lactation period of 21 d, the piglets had no access to the sow’s feed or to creep feed. Sows and piglets were given free access to water throughout the experiment. On the day of farrowing, sows were offered 2.0 kg of the lactation diet, followed by increasing the amount to 3.0 kg daily until ad libitum feeding. The reasons for the sows eliminated from this study were recorded in detail and shown in Supplementary Table S2.

### Measurements of reproductive performance and sample collection

After farrowing, the number and weight of the piglets born, born alive, stillbirths and mummies were recorded, and invalid piglets included stillbirth and mummy. Piglets were weaned at d 21 of lactation.

During sow farrowing, umbilical cords were tied with a short silk line and each piglet was marked with a numbered tag to match the individual piglets with their placentae. After placental expulsion and weight recording, the placentae were collected and snap-frozen in liquid nitrogen (3 to 4 cm from the cord insertion point), and the other fresh placental tissues were immediately fixed in 4% paraformaldehyde. Placental efficiency was calculated by dividing piglet weight by placental weight [17]. In this study, the mean birth weight of the 1,181 piglets was 1.4 ± 0.22 kg (mean ± standard error). The placentae were assigned to two categories according to piglet birth weight: < 1.0 kg (low birth weight, LBW) and 1.4–1.6 kg (normal birth weight, NBW). Litter size at birth was also categorized into 2 classes based on the average number of piglets born alive per litter (15.0 piglets): low yield sow (the number of born alive piglets < 15.0) and high yield sow (the number of born alive piglets ≥15.0).

The blood samples of sows (*n* = 8 per group) were collected in 10 mL centrifuge tubes from the ear vein of the fasted sows at farrowing and at weaning, followed by centrifugation at 3,000×*g* and 4 °C for 15 min to recover the serum. The blood samples of piglets were collected from the anterior vena cava of the piglet whose body weight was closest to the average body weight of the litter at birth (NBW piglets, *n* = 6 per group) on the parturition day and centrifuged at 3,000×*g* and 4 °C for 15 min to recover the serum. At 0.5 h before the birth of the first piglet, colostrum was collected from the functional glands of each sow. Finally, these samples were stored at − 80 °C until further analysis of the oxidative parameters.

### Oxidative stress parameters in serum, colostrum and placenta

The levels of total antioxidant capacity (T-AOC), glutathione (GSH), and malondialdehyde (MDA) were determined using the commercial kits (Nanjing Jiancheng Bioengineering Institute, Nanjing, China) according to the manufacturer’s procedures.

Total protein concentrations in placenta and colostrum were measured according to the instructions of the bicinchoninic acid protein assay kit (Beyotime, Beijing, China). T-AOC, GSH, and MDA in placenta and colostrum were normalized to the total protein. T-AOC is associated with the elimination of free radicals and ROS, blocking peroxidation and thus preventing lipid peroxidation and removing catalytic metal ions, while MDA is the end product of lipid peroxidation and an excellent indicator of oxidative stress [18].

### Placental vascular density

Eight sows were selected from each group, and 12 placenta samples were analyzed, including 6 NBW and 6 LBW placental samples. The number of vessels was determined via image analysis by estimating the average value of 3 slices of one placenta. Briefly, fresh placental tissues fixed in 4% paraformaldehyde were embedded in paraffin and sectioned at 5 μm thickness, followed by staining with hematoxylin and eosin. The area occupied by placental tissues was traced, and the placental vessels in these areas were also traced using a projecting microscope (Olympus CX41, Japan). For each of the 5-μm sections, the total number of vessels in the placental stroma areas were determined, then corrected with the total placental stroma areas measured (per unit area as mm^2^) [10].

### Cell culture and treatments

Porcine vascular endothelial cells (PVECs) were obtained from the Cell Bank of the Chinese Academy of Sciences (Shanghai, China) and cultured in 1,640 medium with 10% fetal bovine serum, 100 U/mL penicillin, and 100 μg/mL streptomycin at 37 °C in 5% CO_2_ atmosphere. The PVECs with cobblestone morphology were passaged at 90% confluence and used for experiments within five passages.

To establish *in vitro* oxidative stress model, H_2_O_2_ was applied to the PVECs. For the H_2_O_2_ treatment experiments, the PVECs were treated with different concentrations (0, 100, 200 or 300 μmol/L) of H_2_O_2_ for 24 h, or 200 μmol/L H_2_O_2_ for 0, 6, 12, 24 or 48 h. After treatments, the PVECs were used for subsequent analysis or treatment.

For the CS treatment experiments, the PVECs were pretreated with various concentrations of CS (0.5, 1 or 2 mmol/L) and/or 5 μmol/L stattic (a selective Stat3 inhibitor) for 2 h, and then challenged with 200 μmol/L H_2_O_2_ for 24 h. After treatments, the PVECs were used for subsequent analysis or treatment.

### Cell viability assay

The PVECs (15,000 cells per well) were seeded in 96-well plates. After treatment as described above, cell viability was measured by cell counting kit-8 assay (CCK-8) (Beyotime) as instructed by the manufacturer. The absorbance of each well at 450 nm was measured using a microplate reader (Bio-Rad Laboratories, Hercules, CA).

### Measurement of intracellular ROS generation

The accumulation of intracellular ROS was examined using the ROS assay kit (Beyotime) according to the manufacturer’s instructions. Briefly, the PVECs (15,000 cells per well) were grown in a 96-well plate and subjected to different treatments as described above. After incubation with 10 μmol/L 2,7-dichlorofluorescein diacetate at 37 °C for 20 min, the fluorescence intensity of the cells was measured using the fluorescence plate reader (BD Falcon, Bedford, MA, USA) at Ex./Em. = 488/525 nm.

### Scratch healing assay

The wound healing scratch assay was used to assess cell migration as previously described [19]. Briefly, cells were seeded onto a 6-well plate and cultured overnight until the formation of a confluent monolayer, followed by making a scratch wound with a 200-μL pipette tip and measuring the effects of H_2_O_2_, CS, and stattic on scratch healing at 24 h after the scratch. The images of the wounded areas were captured using an Olympus inverted microscope and quantified using the ImageJ software.

### In vitro tube formation assay

Matrigel tube formation assays were used to assess the *in vitro* angiogenic activity of PVECs. Briefly, after treatment as described above, PVECs were seeded in 96-well plates precoated with 50 μL Matrigel (BD company, USA) at a density of 1 × 10^4^ cells per well. After 4 h incubation, matrigel-induced morphological changes in PVECs and their tubular networks were photographed at 50 or 100× magnification for analysis using Image J software.

### Trans-well migration assay

The chemotactic migration of PVECs was assayed using a trans-well chamber equipped with a polycarbonate filter with a diameter of 6.5 mm and a pore size of 8 μm. Briefly, after different treaments as described above, PVECs were suspended in 1,640 medium to a final concentration of 5 × 10^4^ cells/ml and were then placed in the upper wells of the chamber, while the lower chamber was filled with 600 μL medium containing 10% FPS. After incubation at 37 °C for 48 h, the cell culture inserts were collected. The cells on the upper side of the filters were removed with cotton-tipped swabs, while the cells on the underside of the filters were fixed with 4% formaldehyde for 30 min, which were stained with crystal violet for 20 min and counted in five randomly chosen fields.

### Quantitative real-time RT-PCR (qRT-PCR) analysis

Total RNA from placenta or PVECs was extracted with the reagent box of Total RNA Kit according to the manufacturer’s instructions. The concentration of RNA was quantified using a NanoDrop_®_ 2000 (Thermo Fisher, USA). After reverse transcription using Primer Script TM RT reagent Kit (Takara, Qingdao, China), qRT-PCR was conducted using SYBR Green on a QuantStudio 6 RealTime PCR System (Thermo Fisher, USA) under the conditions of denaturation at 95 °C for 10 min, amplification at 95 °C for 15 s and 60 °C for 1 min for 40 cycles. Each target gene was individually normalized to the reference gene β-actin by using the quantification method of 2^−ΔΔct^_._ Primers used in this study are shown in Supplemental Table S3.

### Western blotting

Total proteins were extracted from PVECs using the protein extraction kit (Beyotime, Beijing, China) according to the manufacturer’s guide. Briefly, an amount of 10 μg protein was loaded and separated by SDS–PAGE gel electrophoresis, followed by transferring the proteins onto the polyvinylidenedi fluoride membranes (Merck Millipore). After blocking with TBS/T buffer containing 5% milk, the membranes were incubated with the primary antibodies against vascular endothelial growth factor A (VEGF-A) (19003–1-AP, Proteintech, USA, 1:1,000), NADPH oxidase 2 (NOX2) (19013–1-AP, Proteintech, USA, 1:1,000), signal transducer and activator of transcription-3 (Stat3)(ab76315, Abcam, USA, 1:1,500), p-Stat3 (ab68153, Abcam, USA, 1:1,500), and β-actin (4970, CST, USA, 1:1,000). Subsequently, the membranes were incubated with appropriate HRP-conjugated anti-rabbit IgG secondary antibody (AS014, Abclonal, China, 1:5,000). Images were captured using the ChemiDoc MP system (Bio-Rad, Hercules, CA, USA), and band densities were quantified using Image Lab software (Bio-Rad, Hercules, CA, USA) and then normalized to β-actin content.

### Immunofluorescence

Placental tissues fixed in 4% paraformaldehyde were embedded in paraffin and sectioned at 5 μm thickness for platelet endothelial cell adhesion molecule-1 (CD31) immunofluorescence as described previously [19]. The slides were visualized under a fluorescent microscope (Nikon Eclipse C1, Tokyo, Japan). Fluorescence intensities were quantified using ImageJ software (National Institutes of Health, Bethesda, MD).

### Statistical analysis

Data are presented as mean ± SEM and were statistically analyzed using one-way ANOVA and Duncan’s multiple-range test in SPSS 20.0 (SPPS Inc., Chicago). Tamhane’s T2 test was used to assess variance heterogeneity. The stillbirth, LBW, and invalid rates were analyzed using the Chi-square test. Pearson’s correlation coefficient was used to analyze the correlation between piglet birth weight and placental vascular density, T-AOC, and MDA. Additionally, polynomial contrasts were used to evaluate the linear and quadratic effects of CS supplementation on the various parameters measured in the sow experiment. Differences were considered significant at *P* < 0.05, and a tendency was considered at 0.05 ≤ *P* < 0.1.

## Results

### Correlation between birth weight and placental characteristics of piglets

**Fig. 1:**
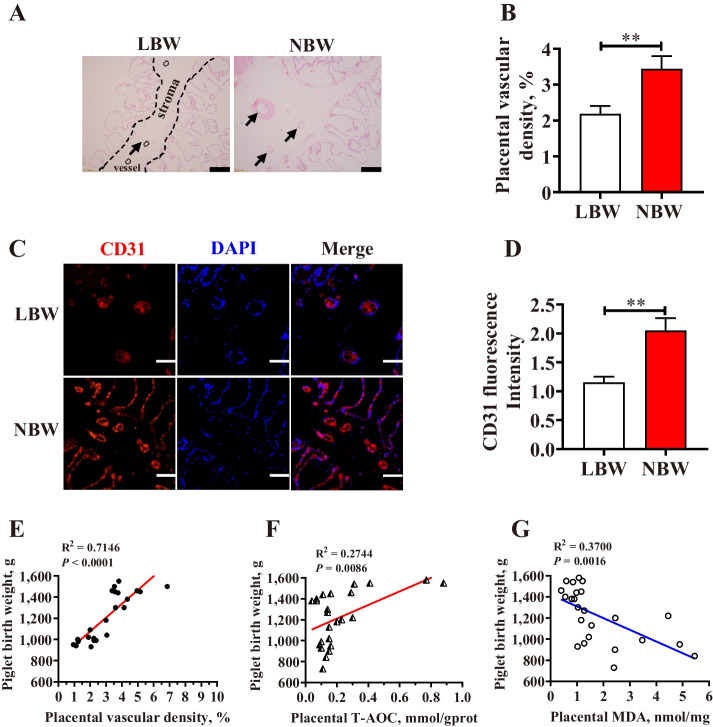
The vessel density distribution in the LBW and NBW placentae. **A**, **B** The hematoxylin and eosin method was used to examine blood vessel density in the LBW and NBW placental tissues, and the black arrows indicate placental blood vessels (bar = 100 μm, *n* = 24). **C**, **D** CD31 immunofluorescence staining in the LBW and NBW placentae (bar = 100 μm). **E** Correlation between placental blood vessel density and piglet birth weight (*n* = 24). **F** Correlation between placental total antioxidant capacity (T-AOC) and piglet birth weight (*n* = 24). **G** Correlation between placental malondialdehyde (MDA) and piglet birth weight (*n* = 24). LBW and NBW indicate piglets with a low birth weight (LBW) (less than 1.0 kg) and normal birth weight (NBW) (1.4–1.6 kg), respectively. Data are presented as mean ± SEM. Differences between groups were considered statistically significant at ** *P* < 0.01

When compared with the NBW placenta, the LBW placenta showed significantly lower (*P* < 0.05) blood vessel density (Fig. 1A, B) and immunostaining intensity of CD31 (biomarker of the endothelial cell in small vessels) (Fig. 1C, D). Meanwhile, piglet birth weight showed a significant positive correlation with placental vascular density (R^2^ = 0.7146, *P* < 0.0001) (Fig. 1E), or T-AOC in placenta (R^2^ = 0.2744, *P* = 0.0086) (Fig. 1F), but a negative correlation with placental MDA level (R^2^ = 0.3700, *P* = 0.0016) (Fig. 1G).

### Reproductive performance

**Fig. 2:**
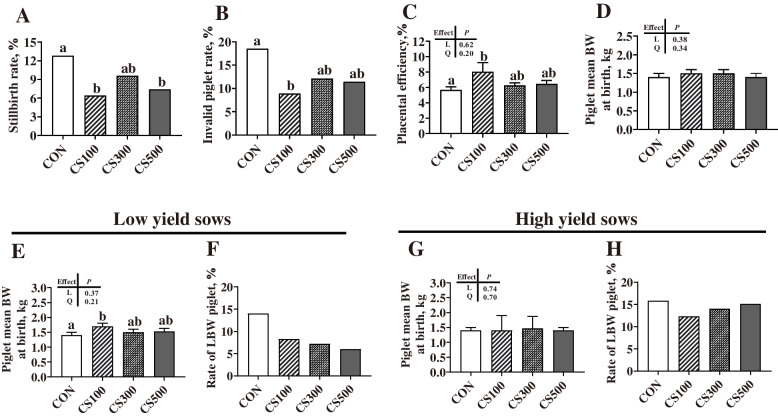
Characteristics of sows and piglets when supplemented with different amounts of cysteamine (CS). **A**, **B** Stillbirth and invalid piglet rates were analyzed using the Chi-square test; **C** Placental efficiency = piglet weight (g)/placental weight (g); **D** Piglet birth weight, with the number of sows being 21, 20, 19 and 19 in CON, CS100, CS300 and CS500, respectively (a-d); **E**, **F** Piglet birth weight and low birth weight (LBW, < 1.0 kg) in low yield sows (i.e., the number of piglets born alive is less than 15), with the number of sows being 11, 6, 8 and 9 in CON, CS100, CS300 and CS500, respectively (e-f); **G**, **H** Piglet birth weight and normal birth weight (NBW) in high yield sows (i.e., the number of piglets born alive is more than 15), with the number of sows being 10, 14, 11 and 10 in CON, CS100, CS300 and CS500, respectively (g-h); CON, basal diet group; CS100/300/500, basal diet supplemented with 100, 300, or 500 mg/kg of CS; Except for rates of stillbirth, invalid piglets, and LBW piglets, all values are expressed as means ± SEM. Linear (L) and quadratic (Q) effects of inclusion amounts of CS were contrasted. Different lowercase letters represent significant difference at *P* < 0.05

The four groups showed significant differences in sow performance (Fig. 2). When compared with the CON group, the CS100 group showed significantly lower (*P* < 0.05) stillbirth and invalid piglet rates, but higher (*P* < 0.05) placental efficiency. In addition, piglet birth weight showed significantly higher (*P* < 0.05) for the low yield sows of the CS100 group versus the CON group.

### Oxidative stress parameters in serum, colostrum and placenta

**Fig. 3:**
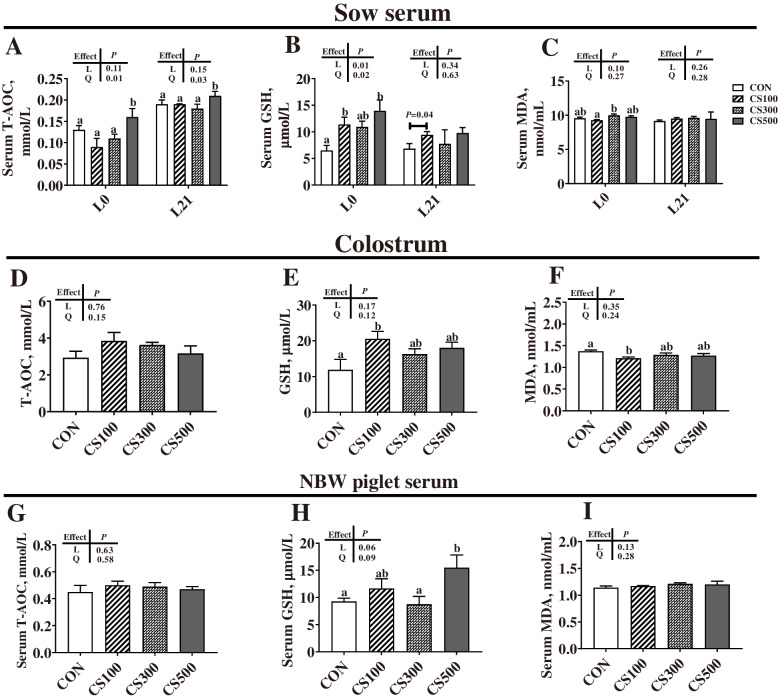
Oxidative stress levels of sows and piglets. **A**, **D**, and **G** T-AOC, total antioxidant capacity; L0, at farrowing; L21, at weaning. **B**, **E**, and **H** GSH, glutathione; **C**, **F**, and **I** MDA, malondialdehyde. CON, basal diet group; CS100/300/500, basal diet supplemented with 100, 300, or 500 mg/kg of cysteamine (CS); NBW indicate piglets with a normal birth weight (1.4–1.6 kg); Data are presented as mean ± SEM (*n* = 6–8). Linear (L) and quadratic (Q) effects of inclusion amounts of CS were contrasted. Different lowercase letters represent significant difference at *P* < 0.05

As shown in Fig. 3A-C, when compared with the CON group, the sows in the CS100 group showed significantly higher (*P* < 0.05) serum GSH levels at farrowing and weaning, and the sows in the CS500 group showed significantly higher (*P* < 0.05) serum T-AOC levels at farrowing and weaning and serum GSH levels at farrowing. For these parameters in colostrum, when compared with the CON group, the CS100 group showed a significantly higher colostrum GSH level but a lower colostrum MDA level (*P* < 0.05) (Fig. 3D-F). Additionally, these oxidative stress parameters in neonatal serum were also investigated (Fig. 3G-I), with a significantly higher (*P* < 0.05) serum GSH level for the NBW piglets in the CS500 group than in the CON group.

**Fig. 4:**
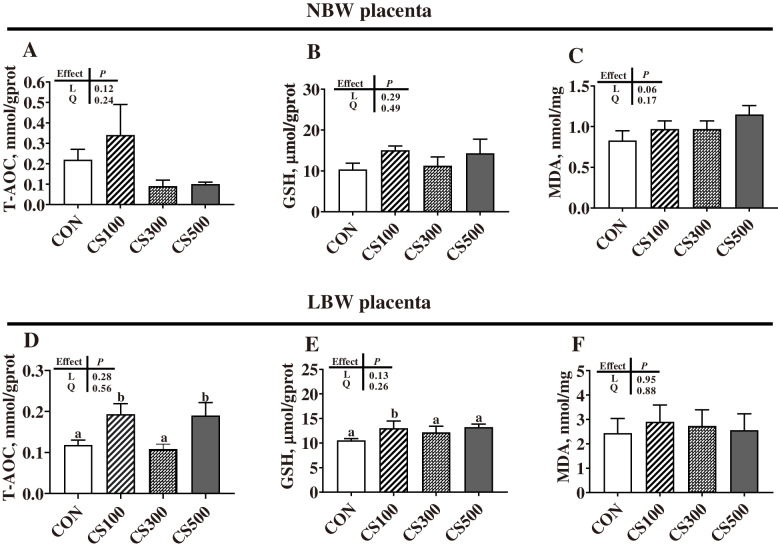
Placental oxidative stress levels. The levels of total antioxidant capacity (T-AOC) (**A**, **D**), glutathione (GSH) (**B**, **E**), and malondialdehyde (MDA) (**C**, **F**) were normalized to the placental total protein. LBW and NBW indicate piglets with a low birth weight (LBW) (less than 1.0 kg) and normal birth weight (NBW) (1.4–1.6 kg), respectively. CON, basal diet group; CS100/300/500, basal diet supplemented with 100, 300, or 500 mg/kg of cysteamine (CS). Data are presented as mean ± SEM (*n* = 6). Linear (L) and quadratic (Q) effects of inclusion amounts of CS were contrasted. Different lowercase letters represent significant difference at *P* < 0.05

Importantly, we observed that the placentae of piglets with different birth weights react differently with maternal CS supply (Fig. 4). Compared with the CON group, the CS100 group showed significantly higher (*P* < 0.05) contents of T-AOC and GSH in the LBW placentae, with no difference observed in the NBW placentae among the four dietary treatments (*P* > 0.05).

**Fig. 5:**
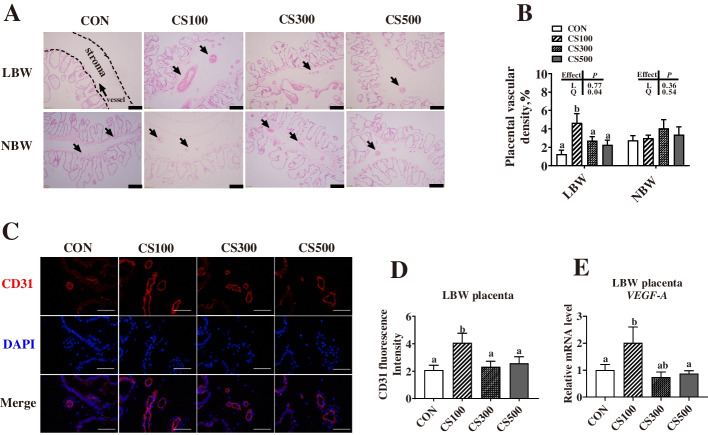
Effects of maternal cysteamine supplementation on the vascular density of placentae. **A**, **B** The hematoxylin and eosin method was used to examine blood vessel density in placental tissues, and the black arrows indicate placental blood vessels (bar = 100 μm). **C**, **D** CD31 immunofluorescence staining in placentae (bar = 50 μm). **E** mRNA relative expression of vascular endothelial growth factor A (*VEGF-A*). CON, basal diet group; CS100/300/500, basal diet supplemented with 100, 300, or 500 mg/kg of cysteamine (CS). LBW and NBW indicate piglets with a low birth weight (LBW) (less than 1.0 kg) and normal birth weight (NBW) (1.4–1.6 kg), respectively. Data are presented as mean ± SEM (*n* = 6). Linear (L) and quadratic (Q) effects of inclusion amounts of CS were contrasted. Different lowercase letters represent significant difference at *P* < 0.05

The effects of CS on placental function were further explored by analyzing the placental vascular density and the expression of angiogenesis-related genes. Figure 5A, B present the effects of CS supplementation on the placental vascular density in LBW or NBW piglets. The placental vascular density showed significantly higher (*P* < 0.05) for the LBW piglets of the CS100 group versus the CON group. For the NBW placentae, the CS supplementation did not significantly change the placental vascular density (*P* > 0.05). Immunostaining analysis also revealed higher (*P* < 0.05) expression levels of CD31 in the LBW placenta in the CS100 group versus the CON group (Fig. 5C, D), and similar results were also found in the mRNA level of *VEGF-A* (*P* < 0.05) (Fig. 5E).

Collectively, dietary 100 mg/kg CS could produce positive effects on the antioxidant capacity and placental angiogenesis of sows.

### Oxidative stress induced by H_2_O_2_ inhibits tube formation and migration *in vitro*

**Fig. 6:**
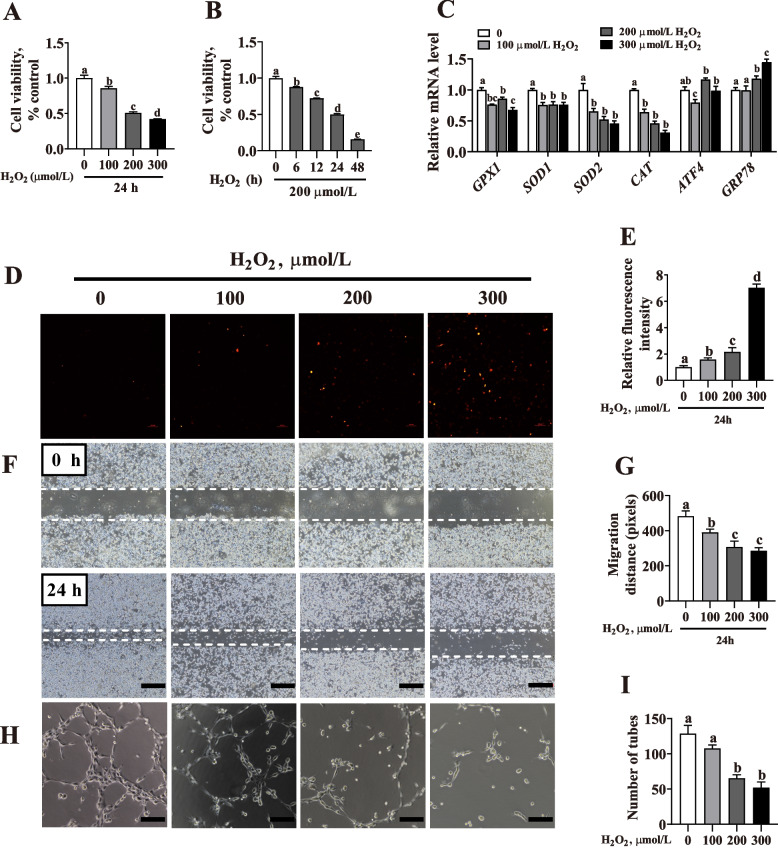
Effects of oxidative stress induced by H_2_O_2_ on the tube formation and migration in porcine vascular endothelial cells (PVECs). **A** PVECs were treated with various concentrations of H_2_O_2_ (0, 100, 200 or 300 μmol/L) for 24 h (*n* = 6). **B** PVECs were treated with 200 μmol/L H_2_O_2_ for 0, 6, 12, 24 or 48 h. CCK8 assay was used to measure cell viability (*n* = 6). **C** The mRNA relative expression of Glutathione peroxidase 1 (*GPX1*), Cu/Zn superoxide dismutase (*SOD1*), Mn-superoxide dismutase (*SOD2*), catalase (*CAT*), activating transcription factor 4 (*ATF4*), and glucose-regulated protein78 (*GPR78*). PVECs were treated with various concentrations of H_2_O_2_ (0, 100, 200 or 300 μmol/L) for 24 h (*n* = 3). **D**, **E** ROS generation in PVECs treated with various concentrations of H_2_O_2_ (0, 100, 200 or 300 μmol/L) for 24 h (*n* = 3). **F**, **G** Scratch healing assay of migratory distance of PVECs treated with various concentrations of H_2_O_2_ (0, 100, 200 or 300 μmol/L) for 24 h (*n* = 3; bar = 500 μm). **H**, **I** Representative images of tube formation by PVECs treated with various concentrations of H_2_O_2_ (0, 100, 200 or 300 μmol/L) for 24 h (*n* = 5; bar = 100 μm). Data are presented as mean ± SEM. Different letters indicate significant differences at *P* < 0.05

The relationship between placental oxidative status and angiogenesis was further investigated by using PVECs to evaluate the effects of oxidative stress on angiogenesis *in vitro*. As shown in Fig. 6A, B, compared with the control group (0 μmol/L H_2_O_2_), the viability of PVECs was significantly decreased (*P* < 0.05) under H_2_O_2_ treatment in a dose- and time-dependent manner. Whether the redox state of PVECs could be changed by H_2_O_2_ was investigated by analyzing the mRNA expression of antioxidant-related genes and the endoplasmic stress markers. The mRNA expression level showed a significant decrease (*P* < 0.05) for *GPX1*, *SOD1*, *SOD2* and *CAT* while a significant increase for *GRP78* (*P* < 0.05) in a dose-dependent manner (Fig. 6C). The ROS level also increased significantly (*P* < 0.05) in a dose-dependent manner (Fig. 6D, E). Meanwhile, tube formation (Fig. 6F, G) and migration (Fig. 6H, I) were significantly impaired (*P* < 0.05) in PVECs after treatment with H_2_O_2_ for 24 h. As 200 μmol/L H_2_O_2_ treatment for 24 h could significantly decrease the viability, tube formation and migration to about 50% and change the redox state of PVECs, this concentration was used for further experiments.

### Oxidative stress impairs the Stat3/VEGF-A pathway in PVECs

**Fig. 7:**
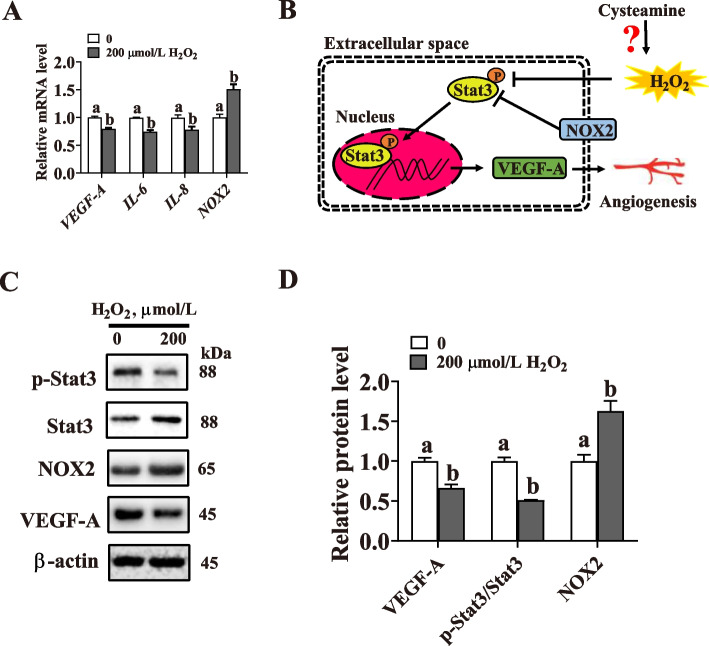
Effects of oxidative stress induced by H_2_O_2_ on the Stat3/VEGF-A pathway in porcine vascular endothelial cells (PVECs). **A** The mRNA expression of angiogenesis-related factors (vascular endothelial growth factor A, *VEGF-A*; interleukin-6/8, *IL-6/8*; NADPH oxidase 2, *NOX2*). **B** Schematic for the mechanism of H_2_O_2_-induced angiogenesis impairment. **C**, **D** Western blotting analysis of the expression of phospho-Stat3 (p-Stat3), NOX2, and VEGF-A. Cells were treated with 200 μmol/L H_2_O_2_ for 24 h. Data are presented as mean ± SEM (*n* = 3). Different letters indicate significant differences at *P* < 0.05

A growing body of evidence supports an important role of Stat3 in placental angiogenesis [20]. In our previous study, Stat3/VEGF-A pathway was found to be impaired in LBW placenta (Hu et al., unpublished data), leading to the question of whether H_2_O_2_-mediated angiogenesis inhibition is controlled by Stat3. After treatment with H_2_O_2_ (200 μmol/L) for 24 h, the relative mRNA expression of Stat3-regulated genes (*IL6*, *IL8*, and *VEGF-A*) in PVECs significantly decreased (*P* < 0.05), in contrast to a significant increase (*P* < 0.05) in the expression of *NOX2* (the upstream negative regulator of Stat3) (Fig. 7A-B). Similar results were obtained from Western blotting analysis (Fig. 7C, D).

### CS rescues H_2_O_2_-induced Stat3 signaling pathway activity

**Fig. 8:**
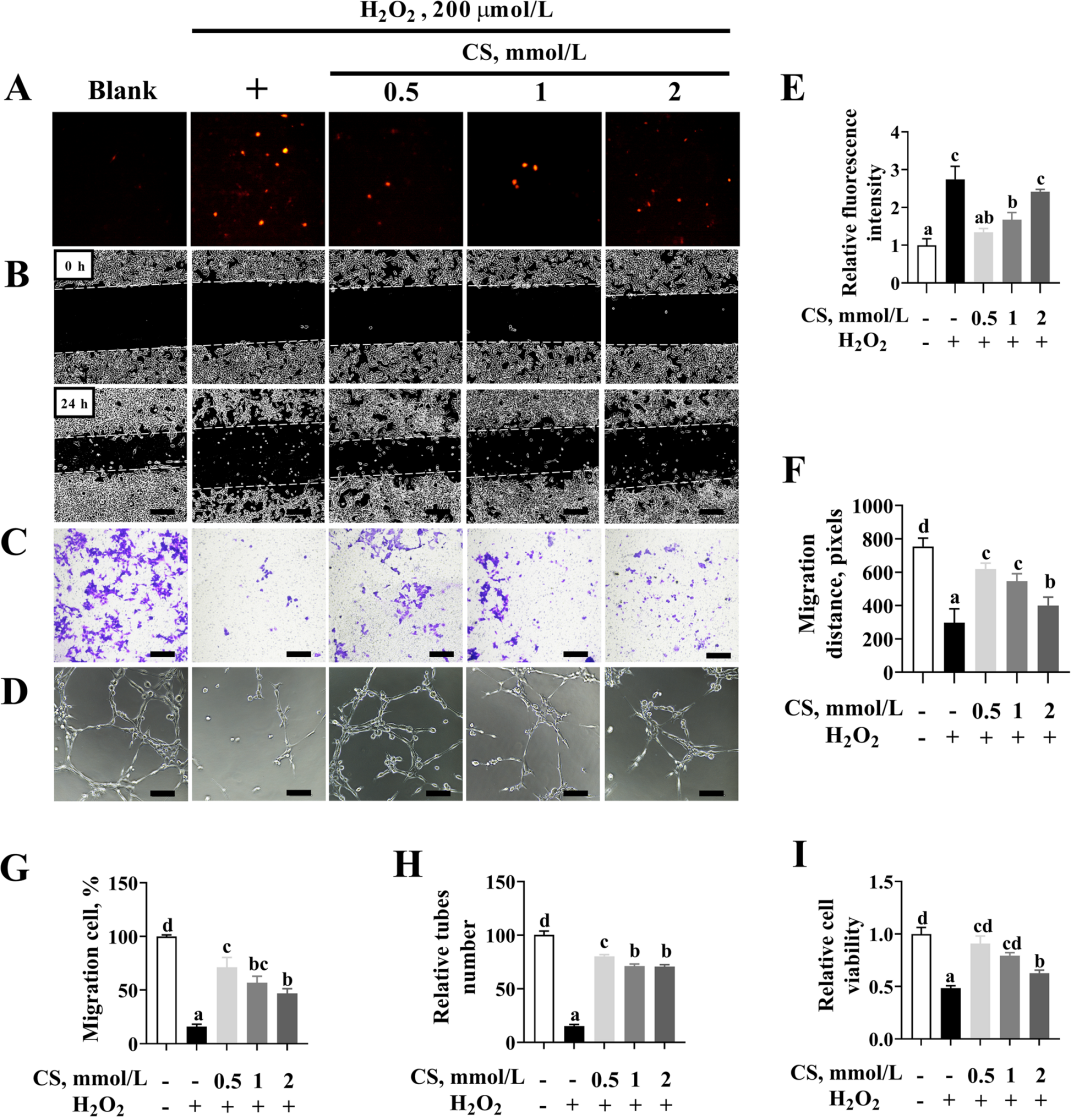
Cysteamine (CS) pretreatment attenuates the effects of H_2_O_2_ on angiogenesis. **A**, **E** The levels of ROS. PVECs were pretreated with various concentrations of CS (0.5, 1 or 2 mmol/L) for 2 h and then treated with 200 μmol/L H_2_O_2_ for 24 h (*n* = 6; bar = 100 μm). **B**, **F** Scratch healing assay of migratory distance. PVECs were pretreated with various concentrations of CS (0.5, 1 or 2 mmol/L) for 2 h and then treated with 200 μmol/L H_2_O_2_ for 24 h (*n* = 3; bar = 500 μm). **C**, **G** Trans-well migration assay of the migratory number of PVECs. After different treatments as described above, PVECs were added to the upper chamber of a trans-well and incubated for 48 h, followed by quantifying PVECs that invaded through the chamber (*n* = 3; bar = 500 μm). **D**, **H** Representative images of tube formation of PVECs after different treatments as described above (*n* = 5; bar = 100 μm). **I** CCK8 assay was used to measure cell viability after different treatments as described above (*n* = 6). Data are presented as mean ± SEM (*n* = 3). Different letters indicate significant differences at *P* < 0.05

The effects of CS on the ROS level and endothelial dysfunction in H_2_O_2_-induced PVECs were also evaluated. As shown in Fig. 8A, E, the ROS level induced by H_2_O_2_ was reduced in the cells pretreated with CS (*P* < 0.05). In addition, the impaired migration by scratch healing assay (*P* < 0.05) (Fig. 8B, F) or by trans-well assay (*P* < 0.05) (Fig. 8C, G), and tube formation (*P* < 0.05) (Fig. 8D, H) induced by H_2_O_2_ were partially recovered in PVECs pretreated with CS (*P* < 0.05). Similarly, H_2_O_2_ resulted in a significant decrease in cell viability, but CS supplementation could alleviate the negative effect of H_2_O_2_ on cell viability (*P* < 0.05) (Fig. 8I).

**Fig. 9:**
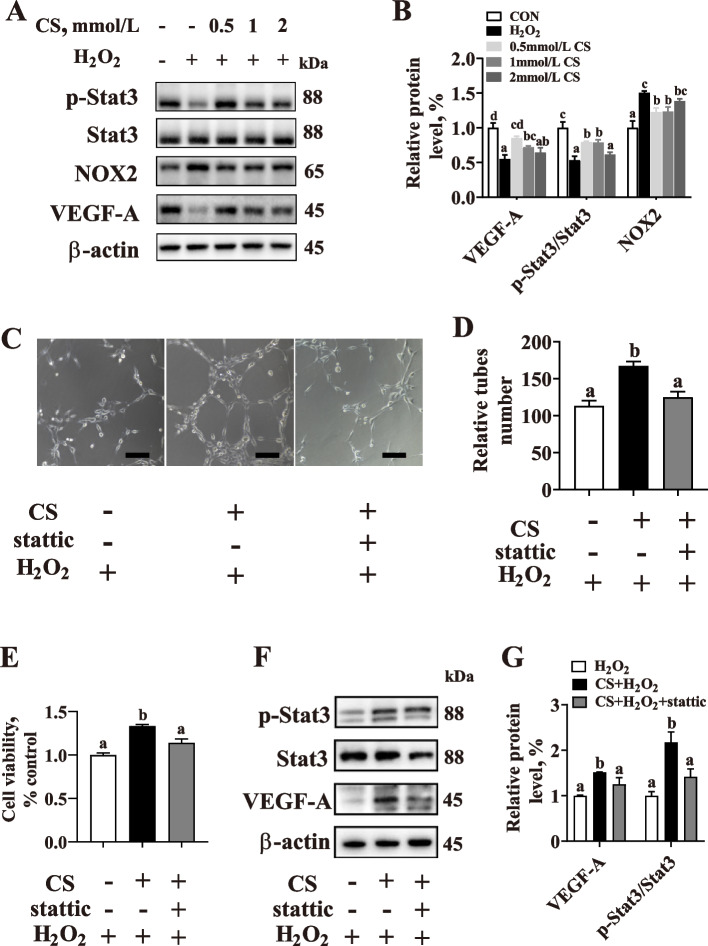
Cysteamine (CS) pretreatment prolongs the phosphorylation of Stat3 in H_2_O_2_-treated PVECs. **A**, **B** Western blotting analysis of the expression of Phospho-Stat3 (p-Stat3), NADPH oxidase 2 (NOX2), and vascular endothelial growth factor A (VEGF-A). Cells were pretreated with various concentrations of cysteamine (0.5, 1 or 2 mmol/L CS) for 2 h, and then challenged with H_2_O_2_ (200 μmol/L) for 24 h (*n* = 3). **C**, **D** Representative images of tube formation by PVECs, pretreated with CS (0.5 mmol/L) and/or inhibitors of Stat3 (5 μmol/L stattic) for 2 h, and then challenged with H_2_O_2_ (200 μmol/L) for 24 h (*n* = 5; bar = 100 μm)5. **E** CCK8 assay was used to measure cell viability after different treatments as described above (*n* = 6). **F**, **G** Western blotting analysis of the expression of p-Stat3 and VEGF-A (*n* = 3). Data are presented as mean ± SEM. Different letters indicate significant differences at *P* < 0.05

Previous studies have shown that CS could activate Stat3 signaling and provide the critical protection required for the survival of intestinal epithelial cells *in vivo* [21]. Therefore, we hypothesized that Stat3/VEGF-A might mediate the enhanced endothelial function in oxidative stress with CS supplementation. The data showed that CS could largely reverse the H_2_O_2_-downregulated Stat3/VEGF-A activity (*P* < 0.05) (Fig. 9A, B). Furthermore, pretreatment with stattic, a selective Stat3 inhibitor, abrogated the effects of CS-mediated pro-angiogenesis (*P* < 0.05) (Fig. 9C, D) and pro-viability (*P* < 0.05) (Fig. 9E) against H_2_O_2_ in PVECs. Meanwhile, inhibition of Stat3 significantly decreased (*P* < 0.05) the cell viability and the VEGF-A protein level in CS pretreated with H_2_O_2_-cultured PVECs (Fig. 9F, G).

The above data confirmed that the Stat3/VEGF-A signaling pathway is invovled in the endothelial protection of CS against oxidative stress.

## Discussion

Previous studies have shown that increased oxidative stress in placenta is associated with the occurrence of adverse pregnancy outcomes, including intrauterine growth restriction and stillbirth [22, 23], suggesting that oxidative stress might contribute to the occurrence of low birth weight and stillbirth. However, the molecular mechanism underlying this link remains unclear. In pigs, high occurrence of intrauterine growth restriction and stillbirth is widely reported [24]. Additionally, pigs are one of the animals most commonly used in biomedical studies on human pregnancy, primarily because of their physiological similarities to humans [25, 26]. The objective of this study was to investigate whether maternal CS supplementation during late gestation improves pregnancy outcomes by examining the changes in antioxidant status and placental angiogenesis. Our study demonstrated that the placentae for LBW neonates were vulnerable to oxidative stress, which might contribute to the occurrence of LBW piglets during pregnancy. However, CS supplementation was shown to have great potential to improve pregnancy outcomes in sows, including birth weight and stillbirth, by reducing oxidative stress and enhancing angiogenesis in placenta.

Placenta plays an important role in fetal growth [27]. Thus, differences in the placental oxidation parameters of piglets with different birth weights were first determined in the present study. We found that birth weight was positively correlated with T-AOC, but negatively correlated with MDA, which was in line with Takagi et al., who reported that oxidative DNA damage was increased in intrauterine growth- restricted placenta and fetus [7]. Excessive ROS could be produced at certain windows in placental development and in some pathological pregnancies, such as those complicated by preeclampsia and/or intrauterine growth restriction, overpowering antioxidant defenses with deleterious outcome [28, 29]. Placental blood vessels are important for fetal growth and development [30], and increased oxidative stress might have a significant effect on placental function, including the proliferation and differentiation of trophoblast cells and vascular reactivity [8, 31]. Likewise, in this study, the LBW placentae showed increased oxidative damage and were vulnerable to angiogenesis impairment. Particularly, oxidative stress induced by H_2_O_2_ (a major type of ROS) prompted intracellular ROS generation and inhibited the tube formation and migration of PVECs as well as the expression of VEGF-A (a major driver for blood vessel formation) *in vitro*. These findings suggest that birth weight is associated with placental redox state, angiogenesis, or both.

Increased oxidative stress during late gestation could lead to a greater percentage of stillbirth [32, 33], which is a substantial cause of economic loss in livestock [34]. Our previous data indicated that alleviating serum oxidative stress at day 109 of gestation contributed to reducing the stillbirth of sows [18]. In this study, CS supply during late gestation was shown to improve the pregnancy outcomes, including birth weight and stillbirth in sows. To test whether the mechanism of CS in improving pregnancy outcomes is related to oxidative stress, we analyzed the effect of dietary CS supplementation on the related parameters in serum and colostrum, and found dietary CS supplementation could partially improve the antioxidant status of sows and their offspring as well as reduce the oxidative stress parameters. Meanwhile, maternal CS supplementation was also shown to partially increase the antioxidant status of placentae for LBW piglets. CS is a known antioxidant and anti-inflammatory agent [35], and previous studies have proposed a number of mechanisms for its protection against oxidative stress. Firstly, CS was reported as a contributor to the cellular redox state of hepatocytes by acting through sulfhydryl-disulfide exchange reactions in cells [35]. Secondly, CS could inhibit nucleotide-binding oligomerization domain-like receptor containing pyrin domain 3 inflammasome by metabolizing cysteine, thereby improving the redox status of maternal-placental interface in sows [11]. Moreover, when used at low concentrations, CS could promote the transport of cysteine into cells, which can be further used to synthesize GSH (one of the most potent intracellular antioxidants) and influence the cellular redox homeostasis [36]. GSH status could reflect the antioxidant capacity of developing embryos. Previous studies have shown that GSH could reduce the formation of ROS and ultimately protect embryos against oxidative stress [37, 38]. In guinea pig, supplementation with GSH precursor N-acetylcysteine was shown to normalize the endothelial function in intrauterine growth-restricted placenta and fetus, thereby normalizing fetal growth [39]. In the present study, the GSH levels were elevated in the LBW placentae of the CS100 group, which benefits the growth and survival of embryo and protects the fetus against ROS damage. In addition, we note that CS supplementation increased the birth weight of piglets only in sows with a low number of piglets, which may be related to their limited uterine capacity in sows with a high number of piglets. Previous reports suggest that at moderate intrauterine crowding, litter size reaches a peak, and further crowding beyond this point could reduce the number of viable embryos/fetuses and weight accumulation, probably by reducing the number of embryos and weight accumulation able to obtain sufficient uterine space for survival due to intrauterine competition for space among embryos [40]. One point we must emphasize is that the litter size is much higher for high yield sows (average 20) than low yield sows (average 13) in this study, which may mask the beneficial effects of CS on high yield sows. The potential mechanism needs to be elucidated in further studies.

Previous reports on the effects of dietary CS levels on redox status and growth performance of animal models, including finishing pigs, sheep and rats, are inconsistent with each other [41]. For instance, previous studies in pregnant rats have shown an increased risk of oxidative stress and IUGR when high doses of CS (150 mg/kg) are applied [41], suggesting the importance of CS dose. In the present study, another important finding is that the dose-effect relationship between CS supplementation and the redox state and pregnancy outcomes is not linear. Compared with the higher dose, the minimum dose (*in vivo* and *in vitro* doses were 100 mg/kg and 500 μmol/L, respectively) was shown as the suitable supplemental dose. A possible explanation is that when used at higher doses, cysteamine oxidation in the presence of transition metals generates H_2_O_2_ molecules, thus causing oxidative stress. In addition, high doses of CS diminish the activity of glutathione peroxidase, the enzyme that catalyzes the oxidation of glutathione to its disulfide [42]. Thus, divergent results of studies using cysteamine could frequently be explained by the antioxidant effect of the drug, which could be counteracted by its direct toxicity when higher doses are used [36]. However, the potential mechanism needs to be revealed in further studies.

The dense blood vessel network in the placenta is responsible for the exchange of respiratory gases, nutrients, and wastes between mother and fetus throughout pregnancy, which is essential for normal fetal growth [43]. Throughout gestation, the vasculature of the placenta is constantly evolving to accommodate the mounting demands of the fetus and could be directly influenced by a number of exogenous factors, such as maternal diet, smoking, oxidative stress, and medication use [44]. Therefore, the effect of CS on the placental vessel density in piglets with different body weights was evaluated in the present study, and 100 mg/kg CS dietary supplementation was found to increase the placental vessel density in LBW piglets. CD31 is a biomarker of endothelial cells in blood vessels, and VEGF-A is a major driver of blood vessel formation [19]. The increased CD31 immunofluorescence intensity and *VEGF-A* mRNA level further demonstrated that the placental vessel density was higher in the CS100 group than in the other three groups. It is worth noting that changes in placental vessel density were consistent with oxidative stress levels only in the LBW placentae among the four groups, further implying that oxidative stress might play an important role in placental angiogenesis. Another key factor for efficient nutrient exchange is placental efficiency [17]. Variations in placental efficiency, a measure of grams of fetus produced per gram of placenta, were initially studied between swine breeds, where increased placental efficiency was found to be associated with larger litters. For instance, when compared to *Yorkshire* pigs, *Meishan* pigs, which are known for large litter sizes, have smaller placentae, but higher vascularity [45]. These observations seem logical in that proper placental vascularization facilitates the efficient exchange of nutrients, implying the importance of improving overall placental efficiency [46], which might also help explain the increased placental efficiency in the CS100 group. Overall, maternal CS supply could decrease the oxidative stress level, thus enhancing angiogenesis in placentae.

Previous studies have reported Stat3 as an important regulator of the adaptive response to oxidative stress in the placenta, which might be related to the increased oxidative stress and the altered trophoblast invasion and placental angiogenesis [44]. The expression of Stat3 and Stat3-mediated genes was shown to be downregulated in our recent study (unpublished data). In the present study, Stat3 targeting genes (*VEGF-A*, *IL-6*, and *IL-8*) and upstream negative regulators (*NOX2*) were used to validate the *in vitro* modulation of H_2_O_2_. The data of our study and others have demonstrated that H_2_O_2_ inhibits the proliferation and migration of PVECs by upregulating NOX2-ROS-mediated inactivation of Stat3 signaling pathway *in vitro* [47], indicating that Stat3 might play an important role in placental angiogenesis in porcine. Previous studies have also shown that CS could activate Stat3 signaling and provide the critical protection required for the survival of intestinal epithelial cells *in vivo* [21]. Here, we found a novel function of CS, modulating the repair of H_2_O_2_-induced PVECs oxidative stress through the Stat3 signaling pathway. The pretreatment of PVECs with statttic (a selective Stat3 inhibitor) and CS failed to prevent the decrease of VEGF-A protein expression and tubification ability induced by H_2_O_2_, strongly suggesting that Stat3 mediates the endothelial protective effect of CS *in vitro*.

## Conclusions

In this study, oxidative stress and impaired angiogenesis were shown to contribute to the occurrence of low-birth-weight piglets during pregnancy, but maternal CS supply at 100 mg/kg during late gestation and lactation of sows could alleviate oxidative stress and enhance angiogenesis in LBW placentae, thereby enhancing pregnancy outcomes, including increased birth weight in low yield sows and reduced stillbirth. The *in vitro* data showed that the underlying mechanism for the positive effects of CS might be related to Stat3 activation in PVECs.

### Supplementary Information

**Additional file 1: Supplementary Table S1.** Composition and nutritional level of gestation and lactation basal diets (Air-Dry Basis, %).

**Additional file 2: Supplementary Table S2.** The number of sows during the experimental period.

**Additional file 3: Supplementary Table S3.** Primer sequences used for real-time PCR.

## Data Availability

The datasets produced and/or analyzed during the current study are available from the corresponding author on reasonable request.
