# Correction: Maternal supply of cysteamine alleviates oxidative stress and enhances angiogenesis in porcine placenta

**DOI:** 10.1186/s40104-023-00955-9

**Published:** 2023-11-24

**Authors:** Shuangbo Huang, Zifang Wu, Zihao Huang, Xiangyu Hao, Longmiao Zhang, Chengjun Hu, Jianfu Wei, Jinping Deng, Chengquan Tan

**Affiliations:** 1 Guangdong Provincial Key Laboratory of Animal Nutrition Control, National Engineering Research Center for Breeding Swine Industry, Institute of Subtropical Animal Nutrition and Feed, College of Animal Science, South China Agricultural University, Guangzhou, 510642 Guangdong China; 2 Guangzhou DaBeiNong Agri-Animal Huabandry Science and Technology Co., Ltd., Guangzhou, 510642 Guangdong China; 3 Guangdong Laboratory for Lingnan Modern Agriculture, South China Agricultural University, Guangzhou, 510642 Guangdong China

**Correction: J Anim Sci Biotechnol 12, 91 (2021)**

**https://doi.org/10.1186/s40104-021-00609-8**

Following publication of the original article [1], the authors reported the images for the 2 mmol/L CS group of the trans-well assay panel in Fig. 8C was incorrectly presented. This error does not affect the conclusion of the study. The correct Fig. 8 should read:

**Fig. 8:**
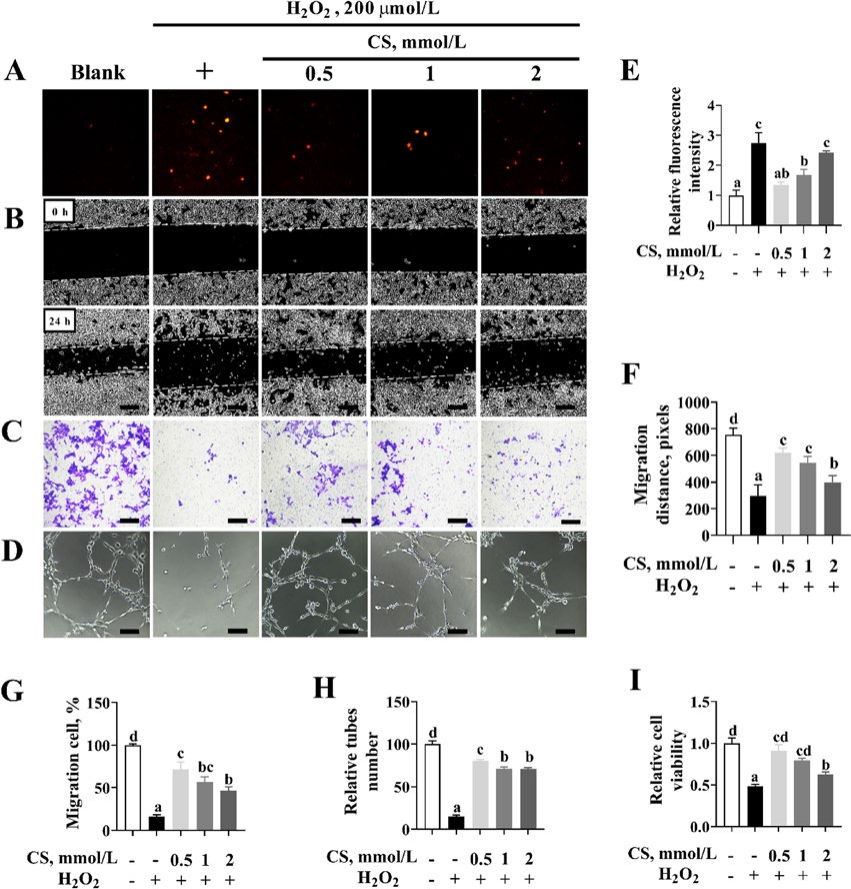
Cysteamine (CS) pretreatment attenuates the effects of H_2_O_2_ on angiogenesis. **A**, **E** The levels of ROS. PVECs were pretreated with various concentrations of CS (0.5, 1 or 2 mmol/L) for 2 h and then treated with 200 μmol/L H_2_O_2_ for 24 h (*n* = 6; bar = 100 μm). **B**, **F** Scratch healing assay of migratory distance. PVECs were pretreated with various concentrations of CS (0.5, 1 or 2 mmol/L) for 2 h and then treated with 200 μmol/L H_2_O_2_ for 24 h (*n* = 3; bar = 500 μm). **C**, **G** Trans-well migration assay of the migratory number of PVECs. After different treatments as described above, PVECs were added to the upper chamber of a trans-well and incubated for 48 h, followed by quantifying PVECs that invaded through the chamber (*n* = 3; bar = 500 μm). **D**, **H** Representative images of tube formation of PVECs after different treatments as described above (*n* = 5; bar = 100 μm). I CCK8 assay was used to measure cell viability after different treatments as described above (*n* = 6). Data are presented as mean ± SEM (*n* = 3). Different letters indicate significant differences at *P* < 0.05

The original article [1] has been updated.
