# Supplementary material for: Maternal supply of cysteamine alleviates oxidative stress and enhances angiogenesis in porcine placenta
Source: J Anim Sci Biotechnol. 2021 Aug 10;12:91. doi: 10.1186/s40104-021-00609-8 (PMC8353810; doi:10.1186/s40104-021-00609-8)
Supplement: Supplementary file 1 — Additional file 1: Supplementary Table S1. Composition and nutritional level of gestation and lactation basal diets (Air-Dry Basis, %). [file 40104_2021_609_MOESM1_ESM.docx]

**Supplementary Table S1.** Composition and nutritional level of gestation and lactation basal diets (Air-Dry Basis, %)

| Item | Gestation ^a^ | Lactation ^b^ |
| --- | --- | --- |
| Ingredient amount, % |  |  |
| corn | 35.8 | 63.1 |
| barley | 30.0 | - |
| wheat-middling | - | 2.5 |
| soya bean meal | 7.5 | 9.5 |
| extruded soybean | - | 6.0 |
| fermented soybean meal | 1.0 | 5.0 |
| beet pulp | 7.0 | - |
| fish meal | - | 2.5 |
| bran | 11.0 | 3.0 |
| corn bran | 2.0 | 2.2 |
| soybean oil | 1.5 | 2.0 |
| stone powder | 1.2 | 1.2 |
| premix | 3.0 | 3.0 |
| total | 100 | 100 |
| Nutritional level |  |  |
| net Energy, kcal/kg | 2178.0 | 2381.0 |
| crude protein, % | 12.9 | 17.4 |
| crude Fiber, % | 5.0 | 2.9 |
| calcium, % | 0.8 | 0.8 |
| non-phytate phosphorus, % | 0.3 | 0.2 |
| lysine, % | 0.1 | 0.3 |

*^a^* Provided by per kilogram of premix: 3500 IU VA, 750 IU VD3, 20 IU VE, 3 mg VK3, 2 mg VB1, 10 mg VB2, 6 mg VB6, 0.03 mg VB12, 50 mg nicotinamide, 30 mg calcium pantothenate, 4 mg folic acid, 0.8 mg biotin.

*^b^* Provided by per kilogram of premix: 2400 IU VA, 750 IU VD3, 40 IU VE, 3 mg VK3, 2 mg VB1, 10 mg VB2, 6 mg VB6, 0.03 mg VB12, 50 mg nicotinamide, 50 mg calcium pantothenate, 4 mg folic acid, 0.8 mg biotin.
