# Supplementary material for: Maternal supply of cysteamine alleviates oxidative stress and enhances angiogenesis in porcine placenta
Source: J Anim Sci Biotechnol. 2021 Aug 10;12:91. doi: 10.1186/s40104-021-00609-8 (PMC8353810; doi:10.1186/s40104-021-00609-8)
Supplement: Supplementary file 2 — Additional file 2: Supplementary Table S2. The number of sows during the experimental period. [file 40104_2021_609_MOESM2_ESM.docx]

**Supplementary Table S2.** The number of sows during the experimental period

| Item | Diet *^a^* | | | |
| --- | --- | --- | --- | --- |
|  | CON | CS100 | CS300 | CS500 |
| at day 85 of gestation | 21 | 21 | 21 | 21 |
| at day 109 of gestation *^b^* | 21 | 20 | 19 | 19 |
| farrowing | 21 | 20 | 19 | 19 |
| culled during lactation *^c^* | 4 | 4 | 3 | 0 |
| weaning | 17 | 16 | 16 | 19 |

*^a^* The four dietary treatments consisted of a gestation or lactation basal diet (CON), or the CON diet supplemented with 100, 300, 500 mg/kg cysteamine (CS100, CS300, and CS500).

*^b^* 5 sows were culled because of unqualified feed intake or sickness.

*^c^* 11 sows were culled because of poor milk.
