# Supplementary material for: Maternal supply of cysteamine alleviates oxidative stress and enhances angiogenesis in porcine placenta
Source: J Anim Sci Biotechnol. 2021 Aug 10;12:91. doi: 10.1186/s40104-021-00609-8 (PMC8353810; doi:10.1186/s40104-021-00609-8)
Supplement: Supplementary file 3 — Additional file 3: Supplementary Table S3. Primer sequences used for real-time PCR. [file 40104_2021_609_MOESM3_ESM.docx]

**Supplementary Table S3.** Primer sequences used for real-time PCR

| Genes *^a^* | Direction | Primers Sequences (5’ to 3’) | Accession | Size (bp) |
| --- | --- | --- | --- | --- |
| *GPX1* | Forward | CCTCAAGTACGTCCGACCAG | NM_214201.1 | 85 |
|  | Reverse | GTGAGCATTTGCGCCATTCA |  |  |
| *SOD1* | Forward | CATTCCATCATTGGCCGCAC | NM_001190422.1 | 118 |
|  | Reverse | TTACACCACAGGCCAAACGA |  |  |
| *SOD2* | Forward | GGACAAATCTGAGCCCTAACG | NM_214127.2 | 159 |
|  | Reverse | CCTTGTTGAAACCGAGCC |  |  |
| *CAT* | Forward | CGAAGGCGAAGGTGTTTG | XM_021081498.1 | 374 |
|  | Reverse | AGTGTGCGATCCATATCC |  |  |
| *ATF4* | Forward | AACATGGCCGAGATGAGCTTCC | XM_021090887.1 | 256 |
|  | Reverse | TCTCCACCATCCAGTCTGTCCC |  |  |
| *GRP78* | Forward | AGTCCCGCAGATTGAAGTCA | XM_001927795.7 | 132 |
|  | Reverse | TCTTCAGGTGTCAGGCGATT |  |  |
| *NOX2* | Forward | TGTATCTGTGTGAGAGGCTGGTG | NM_214043.2 | 156 |
|  | Reverse | CGGGACGCTTGACGAAA |  |  |
| *IL-6* | Forward | GGCAAAAGGGAAAGAATCCAG | NM_001252429.1 | 87 |
|  | Reverse | CGTTCTGTGACTGCAGCTTATCC |  |  |
| *IL-8* | Forward | GCTCTCTGTGAGGCTGCAGTTC | NM_213867.1 | 79 |
|  | Reverse | AAGGTGTGGAATGCGTATTTATGC |  |  |
| *VEGF-A* | Forward | CCTCGGAGCGGAGAAAGCAT | NM_214084.1 | 126 |
|  | Reverse | TGTCACATCTGCAAGTACGTTCG |  |  |
| *β-actin* | Forward | CACCTTCTACAACGAGCTGC | XM_021086047.1 | 95 |
|  | Reverse | TCATCTTCTCACGGTTGGCT |  |  |

*^a^ATF4*: activating transcription factor 4; *CAT*: catalase; *GPX1*: Glutathione peroxidase 1; *GPR78*: glucose-regulated protein78; *NOX2*: NADPH oxidase 2; *SOD1*: Cu/Zn superoxide dismutase; *SOD2*: Mn-superoxide dismutase; *β-actin*: actin beta; *IL-6/8*: interleukin- 6/8; *VEGFA*: vascular endothelial growth factor A.
